# Testing Deprivation and Threat: A Preregistered Network Analysis of the Dimensions of Early Adversity

**DOI:** 10.1177/09567976221101045

**Published:** 2022-09-08

**Authors:** Sofia Carozza, Joni Holmes, Duncan E. Astle

**Affiliations:** 1 MRC Cognition and Brain Sciences Unit, University of Cambridge; 2 School of Psychology, University of East Anglia

**Keywords:** early adversity, deprivation, threat, child development, network analysis, Avon Longitudinal Study of Parents and Children (ALSPAC), open materials, preregistered

## Abstract

Despite abundant evidence of the detrimental effects of childhood adversity, its nature and underlying mechanisms remain contested. One influential theory, the *dimensional model of adversity and psychopathology*, proposes deprivation and threat as distinct dimensions of early experience. In this preregistered analysis of data from the Avon Longitudinal Study of Parents and Children (ALSPAC), we used a network and clustering approach to assess the dimensionality of relationships between childhood adversity and adolescent cognition and emotional functioning, and we used recursive partitioning to identify timing effects. We found evidence that deprivation and threat are separate dimensions of adversity and that early experiences of deprivation cluster with later measures of cognition and emotional functioning. This cluster varies by age of exposure; it includes fewer forms of deprivation as children grow from infancy to middle childhood. Our measures did not form a specific cluster linking threat to emotional functioning.

Growing up in an adverse environment—such as chronic poverty or an abusive household—can severely constrain a person’s capacity to thrive throughout life (Bellis et al., 2019). Yet it has been challenging to develop a clear and coherent account of how early adversity alters the course of development. To date, studies of early adversity have largely fallen into two camps. The first posits that each exposure affects development through a specific pathway. However, this is hard to reconcile with shared common features and high rates of co-occurrence across both adversities and outcomes (Kessler et al., 1997; McLaughlin et al., 2021). The second approach assumes that all adversities act together, regardless of their type, through accumulating stress (Evans et al., 2013). Although parsimonious, this view is hard to reconcile with the qualitative variability among exposures and the heterogeneity of the biological and psychosocial processes they likely engage (McLaughlin et al., 2021). Finally, both approaches rarely consider the timing of childhood adversity, despite the potential importance of sensitive periods of development (Knudsen, 2004). This is especially true of cross-sectional studies, which tend to assess adversity once through retrospective self-report, a method both imprecise and subject to bias (Susser & Widom, 2012).

One promising path to clarity comes from the *dimensional model of adversity and psychopathology* (henceforth “the dimensional model”), which identifies two core domains of early experiences of adversity: threat and deprivation (McLaughlin et al., 2014). *Threat* is the risk or realization of harm to a child’s survival, whereas *deprivation* is the absence of expected environmental input. The model proposes that, by changing the course of experience-dependent neurodevelopmental processes, threat alters socioemotional functioning and deprivation constrains learning and cognitive control (Lambert et al., 2017). Given the unique and protracted trajectories of the implicated processes (Tierney & Nelson, 2009), the model can accommodate the importance of the timing of the exposure. Importantly, although many children experience both deprivation and threat, individual forms of adversity often involve one or the other (McLaughlin et al., 2014); it is therefore possible to measure the dimensions independently and potentially disentangle their mechanisms and outcomes. Thus, the dimensional model avoids the dual pitfalls of a narrow-specificity approach and a reductive cumulative-risk approach.

There is widespread support for this model. Children who experience threat early in life are more likely to exhibit emotional dysregulation, including altered emotional expression, heightened emotional reactivity, and decreased emotional understanding (Dvir et al., 2014), whereas children who experience deprivation tend to show later cognitive difficulties, a relationship mediated by lower complex cognitive stimulation (Rosen et al., 2020). More recently, studies have explicitly tested the dimensional model and discovered findings that largely—though not unequivocally—align with its predictions. For instance, Machlin et al. (2019) found associations between threat and fear learning and between deprivation and deficits in cognitive control (see also Lambert et al., 2017; Miller et al., 2018; Sumner et al., 2019).

However, the interpretation of these studies as support for the dimensional model has its limitations. Most categorize experiences as deprivation or threat a priori before subsequently implementing them as independent predictors of outcomes and therefore cannot accommodate more complex relationships between forms of adversity and developmental differences. Furthermore, studies often fail to control for co-occurring adversity (e.g., Bos et al., 2009) or rely on a single imperfect measure to do so (e.g., Dennison et al., 2019), thereby remaining vulnerable to *residual confounding*, in which spurious associations appear to be significant predictors because of measurement error (Bignardi et al., 2022). In view of these risks, the use of robust multivariate methods could provide important additional support for the dimensionality of adversity.

**Statement of Relevance**

Children who experience adversity early in life—such as chronic poverty or abuse—face numerous obstacles to lifelong flourishing. To effectively mitigate the burden of adversity, it is critical to build a strong scientific account of how it affects human development. In this paper, we assess the *dimensional model of adversity and psychopathology*, a prominent theory of early adversity that distinguishes experiences of threat from experiences of deprivation. Disentangling threat (which is thought to interfere with emotional regulation) from deprivation (which may constrain cognitive development) would enhance efforts to protect children from the effects of adversity. Our study—which includes a large population-based sample and prospective measures—found that although threat and deprivation are distinct dimensions of adversity, deprivation shows broad and nonspecific links with later cognition and emotion. These findings highlight the critical importance of methodologically diverse studies in triangulating the impact of early adversity on child development.

One recent and notable example of such an approach comes from Sheridan et al. (2020), who used network analysis to evaluate the dimensional model. The authors first fitted network models to measures of childhood adversity, cognition, and emotional function and then used consensus clustering to identify communities of nodes (i.e., clusters of measures)—a data-driven approach that required no prior assumption of dimensionality. Across two samples, cognition clustered with measures of socioeconomic status, and emotional reactivity clustered with physical neglect, abuse, and violence exposure. Overall performance on an emotional Stroop task clustered with deprivation in one sample and threat in the other. Although these clusters do not perfectly align with deprivation and threat, the dimensional model outperformed the cumulative risk model in a subsequent hypothesis-testing procedure. This groundbreaking study was the first to use network analysis to explore adversity and provides critical evidence for the dimensional model.

There are several key ways to further advance the network approach. First, the concurrent measurement of past adversity and current well-being used by Sheridan et al. (2020) may exaggerate associations (Newbury et al., 2018), a risk that could be avoided by using longitudinal prospective data. Second, because network accuracy improves with increasing sample size (Epskamp et al., 2018), using a larger sample would permit greater confidence in the characterization of the dimensional structure of adversity. Third, given that neglect was consistently clustered with threat in Sheridan et al. (2020), including additional forms of psychosocial deprivation (such as parental separation) would test the proposed breadth of this dimension. Finally, testing for a moderating effect of timing of exposure may draw out age-related differences in the network structure of adversity.

In this study, we evaluated the dimensional model by applying a network approach to data from a large longitudinal birth cohort, the Avon Longitudinal Study of Parents and Children (ALSPAC; Boyd et al., 2013; Fraser et al., 2013). We fitted mixed graphical models to prospective measures of adversity and measures of cognition and emotional functioning in adolescence. We then identified communities of nodes and searched for an effect of the timing of adversity on the network structure using recursive partitioning. We made the following predictions (https://aspredicted.org/dp8r5.pdf). First, a network approach applied to early adversity in a longitudinal cohort study with rich measures of early life experience will support the dimensional model, with deprivation and threat-related adversities clustering in two communities that respectively cluster with cognitive and emotional outcomes. Second, the age at which children experience early adversity will alter the dimensional clustering of adversity with later cognitive ability and emotional functioning. Third, over the course of childhood, different experiences will vary in their relative importance for the dimensional structure of adversity and psychopathology.

## Method

### Participants

We utilized data from ALSPAC, a prospective population-based cohort study (Boyd et al., 2013; Fraser et al., 2013). ALSPAC recruited pregnant women residing in Avon, United Kingdom, with expected delivery dates between April 1, 1991, and December 31, 1992. Of 14,541 initial pregnancies, 14,062 infants were alive at birth and 13,988 one year later. The study continues to follow these women, their partners, and their children. The current analysis uses data from maternal questionnaires completed during the first 7 years of the child’s life as well as a maternal questionnaire and neuropsychological tasks completed by the child during adolescence. A timeline of data collection is available in Table S1 in the Supplemental Material available online. Further details about the variables are available in a fully searchable data dictionary on the study website (http://www.bristol.ac.uk/alspac/researchers/our-data/).

### Ethics statement

Ethical approval for the study was obtained from the ALSPAC Ethics and Law Committee and from the local research ethics committees. Informed consent for the use of data collected via questionnaires and clinics was obtained from participants following the recommendations of the ALSPAC Ethics and Law Committee at the time. The ALSPAC Executive Committee approved this study, which consists of secondary analysis of fully anonymized ALSPAC data.

### Measures

#### Early adversity

Maternal self-report questionnaires from five time points across childhood were used to identify exposure to 10 forms of early adversity. The study questions for each variable are listed in Table S2 in the Supplemental Material. Eight exposures are binary variables: emotional domestic violence, physical domestic violence, parental physical cruelty, parental emotional cruelty, sexual abuse, physical abuse, a change of primary caregiver, and prolonged parental separation. At each time point, each of these exposures was coded as having occurred if the mother reported that her child had experienced it since the previous time point. Three exposures are composite scores of multiple questions: financial difficulties, maternal-caregiver neglect, and paternal-caregiver neglect. The financial-difficulties score is the sum of ratings of how difficult the mother found it to afford food, clothing, heating, rent or mortgage, and items for the child at that time. The scores for maternal- and paternal-caregiver neglect are the additive inverse of the sum of how frequently the mother and her partner engaged in a range of activities with the child, such as feeding or playing. All variables used the same questions across time points except for the scores for maternal- and paternal-caregiver neglect, which included more questions at later ages to account for a wider range of caretaking activities. The maternal-neglect, paternal-neglect, and financial-difficulties scales demonstrated adequate internal consistency across time points, with respective Cronbach’s αs falling between 0.54 and 0.77, 0.81 and 0.88, and 0.88 and 0.90.

#### Cognition

Children completed cognitive assessments in adolescence. The vocabulary and matrix reasoning subscales of the Wechsler Abbreviated Scale of Intelligence (WASI) were administered at age 15.5 years (Wechsler, 1999). The current study utilized the total IQ score, approximated from the age-adjusted standardized subscale scores, as a measure of general intelligence. A stop-signal task (SST) was also administered at age 15.5 (Logan et al., 1984). Participants were presented with a series of visual stimuli, either the letter “X” or the letter “O,” and asked to press the corresponding button on a stimulus box unless a bleep (the stop signal) sounded after the presentation of the letter. As in previous studies (Donati et al., 2019), the sum of correct trials (out of 32) was used as a measure of inhibitory control. Finally, a computerized *N*-back task was administered at age 17.5 (Kirchner, 1958). Participants were presented with a series of numbers and asked to respond by pressing “1” whenever a number occurred that was identical to the number *N* trials before, and “2” in all other cases. Participants completed 48 trials of both two-back and three-back conditions. To account for possible response bias, we calculated the discrimination index *d*′ by subtracting the *z* score of the false-alarm rate from the *z* score of the hit rate, where the hit rate is the proportion of targets correctly identified as targets and the false-alarm rate is the proportion of nontargets falsely identified as targets. As in previous studies (e.g., Haatveit et al., 2010), *d*′ from the two-back condition was used as a measure of working memory.

#### Emotional functioning

Mothers completed the short form of the parent Strengths and Difficulties Questionnaire (SDQ) when the children were 16 years old. The SDQ consists of 25 items about the child during the previous 6 months, to which the adult responds on a 3-point scale (Goodman, 1997). The psychometric properties of the SDQ are well established (Muris et al., 2003). Four of the five subscales were used in the current study: conduct problems, hyperactivity/inattention, emotional symptoms, and peer-relationship problems. In line with previous work (Goodman et al., 2010), the conduct and hyperactivity/inattention subscales were summed as a measure of externalizing problems, whereas the emotional and peer-relationship problems subscales were summed as a measure of internalizing problems.

### Missing data and outliers

As is the case with most longitudinal studies, ALSPAC suffers from selective attrition and missing data, which can weaken generalizability and result in the underestimation of adverse outcomes (Graham, 2009; Wolke et al., 1995). However, systematic dropout does not necessarily bias findings. In fact, a previous analysis of ALSPAC data found that attrition did not alter the association between early environmental risk and a later psychiatric diagnosis (Wolke et al., 2009). Furthermore, missing data can be estimated without introducing bias as long as missingness is either unrelated to any other variables or related only to observed variables (i.e., missing completely at random or missing at random; Little & Rubin, 2002).

In the current study, rates of missingness increased from one time point to the next, from 18% to 21% for variables in infancy to 61% to 76% for variables in adolescence. Following the preregistration, we excluded participants who lacked data for more than 30% of variables (*n* = 5,500), dropped out of the study before reaching adolescence (*n* = 6,961), or scored 3 or more standard deviations above or below the mean on continuous variables (*n* = 218). The first two criteria minimized the error introduced by imputing data at higher rates of missingness (Tang & Ishwaran, 2017), whereas the third eliminated extreme and unreliable scores that can arise through human error (Osborne & Overbay, 2004). Thus, 5,812 children were retained for analysis. Variables were excluded from the analysis if they were missing in more than 30% of the retained sample in order to minimize imputation error (Tang & Ishwaran, 2017), which could bias clustering results. One variable, the working memory task, met this threshold.

Retained participants lacked an average of 4.4% of data. To establish the plausibility of assuming our data were missing at random, we used the *finalfit* package in R (Version 4.0.4, “Lost Library Book”), which checks for predictors of missingness using Kolmogorov-Smirnoff and two-sided χ^2^ tests (Harrison et al., 2020). After Bonferroni correction for multiple comparisons, participants who lacked complete data were more likely to have experienced domestic violence, a change in primary caregiver, maternal and paternal neglect, and financial difficulties.

Missing data for all retained participants were imputed using a random-forest algorithm, as implemented in the *MissForest* package (Stekhoven & Bühlmann, 2011). The algorithm consists of an iterative process of training and prediction, which continues until the difference between the new imputation and the previous imputation begins to rise for both categorical and continuous variables. The algorithm demonstrates greater accuracy than other imputation methods, accommodates mixed data types, and does not require tuning (Tang & Ishwaran, 2017; Waljee et al., 2013). The method also produces unbiased estimates of the accuracy of the imputation (Breiman, 2001). Our estimates were near zero—which corresponds to perfect performance—for both continuous and categorical variables (normalized root-mean-square error = .0016, proportion of falsely classified entries = .0352). Similar descriptive statistics (see Table S3 in the Supplemental Material) and correlations (see Fig. S1 in the Supplemental Material) were observed in the full sample, the project sample before imputation, and the project sample after imputation.

To further evaluate the representativeness of our findings and whether they were influenced by our exclusion criteria, we repeated the imputation and network analysis for all participants and variables (i.e., disregarding our exclusion criteria). The results were nearly identical to the findings reported below and can be found in Figures S2, S3, and S4 in the Supplemental Material.

### Statistical analyses

Analyses were carried out in R (Version 4.0.4, “Lost Library Book”) using code that is available on OSF (https://osf.io/y6f2c/).

#### Variable preparation

First, we summed each adversity measure across all five time points to obtain a total score for each form of adversity. The standardized *z* score was taken of continuous measures. Then the following network estimation and analysis procedure was performed on three sets of variables: all measures, measures of adversity only, and measures of emotional functioning and cognition only.

#### Network estimation and visualization

We fitted mixed graphical models to the data using the *mgm* package (Haslbeck et al., 2021). This method characterizes relationships between variables of mixed data types using node-wise regression. Only pairwise relationships were considered. To minimize spurious edges, we regularized networks using a least absolute shrinkage and selection operator (LASSO; Tibshirani, 1996). The tuning parameter λ, which determines the severity of the penalty applied to weak edges, was chosen using the extended Bayesian information criterion (EBIC; Foygel & Drton, 2010). Following previous work, we set the hyperparameter γ to 0.25 (Hevey, 2018). Edges were retained if both parameters were nonzero. Networks were visualized with the *qgraph* package (Epskamp et al., 2012).

#### Community detection

We identified densely connected groups of nodes using the Walktrap algorithm (Pons & Latapy, 2006) as implemented in the *igraph* package (Csárdi & Nepusz, 2005). The algorithm uses random walks to derive a measure of distance between nodes and then iteratively merges nodes into communities before selecting the partition that maximizes modularity. Walktrap has been reported to uncover the true network structure under a range of conditions, including networks with fewer nodes (Yang et al., 2016). The strength of clustering was evaluated using the modularity index *Q*, which quantifies the difference between the observed intracommunity connectivity and that expected by chance (Newman, 2004).

#### Centrality measures

The strength of each node, or the sum of the absolute weights of every edge, was calculated using the *qgraph* package (Epskamp et al., 2012). The bridge strength of each node, or the sum of the absolute weights of edges that cross clusters, was calculated using the *networktools* package (Jones, 2020). The stability of these parameters was assessed using the case-dropping bootstrap procedure in the *bootnet* package (Epskamp et al., 2018). This method generates subsamples by dropping a given percentage of the original sample without replacement and then reestimating parameters. Stability is quantified using the correlation-stability (CS) coefficient, which indicates the proportion of the sample that can be dropped while the parameter retains, with 95% probability, a 0.7 correlation with its original value. We generated 2,000 subsamples, consisting of 2.5 to 97.5% of the data set.

#### Recursive partitioning

To test for an effect of the timing of adversity, we used model-based partitioning (Zeileis et al., 2008) as implemented in the *networktree* package (Jones et al., 2020). This method first assesses the instability of parameters with respect to a partitioning variable and then identifies the optimal split points that maximize the heterogeneity of the covariance structures in the daughter nodes. The procedure demonstrates sufficient power to detect medium-to-large effects in adequately large samples (Jones et al., 2020). After splitting the data by the age at which adverse experiences occurred, we repeated our network-estimation approach to obtain subnetworks, clusters, and centrality measures for each segment of childhood. To isolate the unique effects of adversity in each segment, we deviated from our preregistered analysis by controlling for exposure to adversity at other ages. We accomplished this by computing a sum of the adversities that occurred in the other two stages of childhood and implementing it as a moderator in each node-wise regression.

## Results

### Descriptive statistics

**Fig. 1.**
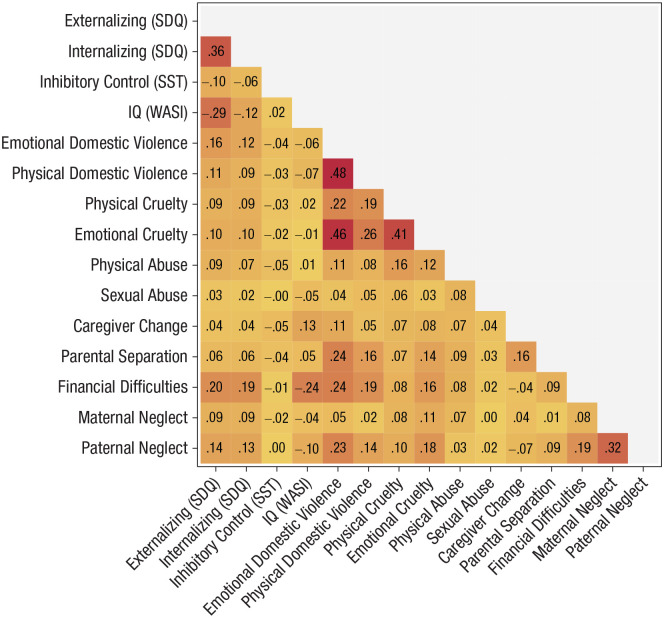
Spearman correlations between network variables. Red indicates stronger positive associations. SDQ = Strengths and Difficulties Questionnaire; SST = stop-signal task; WASI = Wechsler Abbreviated Scale of Intelligence.

Spearman correlations between network variables are presented in Figure 1. This includes measures of cognitive and emotional functioning in adolescence as well as adversities summed across all of childhood. Descriptive statistics for these variables can be found in Table S3, whereas statistics for adversities broken down by individual time point can be found in Table S4. Because no participants reported sexual abuse at two time points, regressions could not be conducted, and the variable was excluded from the analysis.

### Network estimation and parameters

**Fig. 2.**
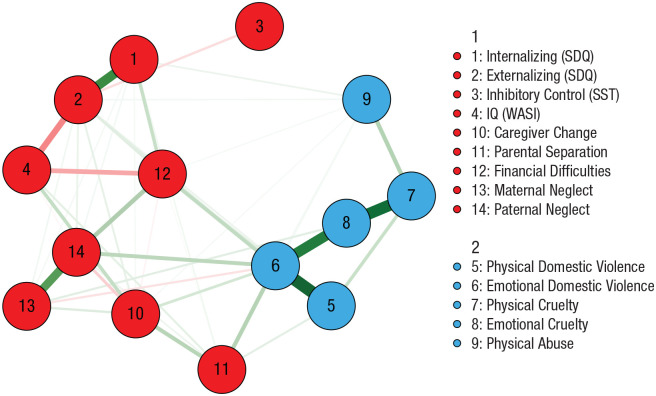
Network of summed adversities across childhood and adolescent emotional functioning and cognition. The color of each node denotes its group membership, and the thickness of each edge denotes its strength. Green and red edges correspond to positive and negative relationships of conditional dependence, respectively. SDQ = Strengths and Difficulties Questionnaire; SST = stop-signal task; WASI = Wechsler Abbreviated Scale of Intelligence.

The overall network, estimated using the sums of each form of adversity across childhood, is shown in Figure 2. Two interrelated clusters of nodes within the network were identified. The first group consisted of all threat-related forms of adversity, whereas the second group consisted of all deprivation-related forms of adversity together with measures of adolescent emotional functioning and cognition. The modularity of the clustering solution was low to moderate (*Q* = 0.30). Analysis of the full sample produced the same clusters, with the exception that parental separation was included in the threat group (see Fig. S2).

The strongest nodes were emotional domestic violence and emotional cruelty, followed by physical domestic violence and physical cruelty (see Fig. S5 in the Supplemental Material). The parental-separation and financial-difficulties nodes exhibited the highest bridge strength, indicating their relatively strong connections to the threat cluster. The bootstrapping procedure indicated excellent stability, with high CS coefficients for node strength (0.92) and bridge strength (0.83; see Fig. S6 in the Supplemental Material).

**Fig. 3.**
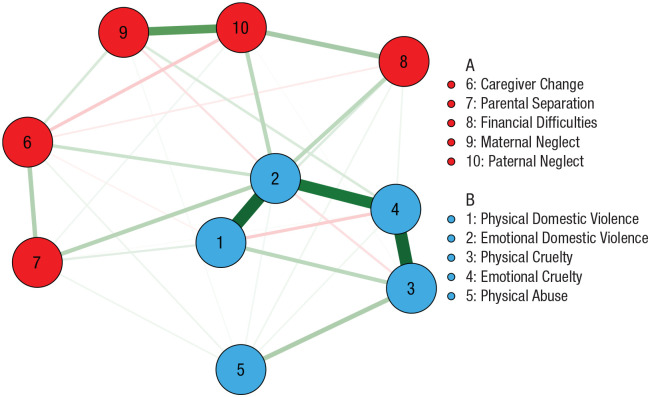
Network of summed adversities across childhood. The color of each node denotes its group membership, and the thickness of each edge denotes its strength. Green and red edges correspond to positive and negative relationships of conditional dependence, respectively.

To test the dimensionality of early experiences without the influence of later cognitive and emotional functioning, we constructed a network on adversity variables alone. As shown in Figure 3, two clusters emerged: one of deprivation-related nodes and one of threat-related nodes. The modularity of the network was low (*Q* = 0.21), but the bootstrapping procedure indicated highly stable node strength (CS = 0.96) and bridge strength (CS = 0.81; see Fig. S6 in the Supplemental Material). Analysis of the full sample produced the same clusters (Fig. S3).

No clusters were present in the network of emotional functioning and cognition measures alone (see Fig. S7 in the Supplemental Material).

### Recursive partitioning and subnetwork estimation

**Fig. 4.**
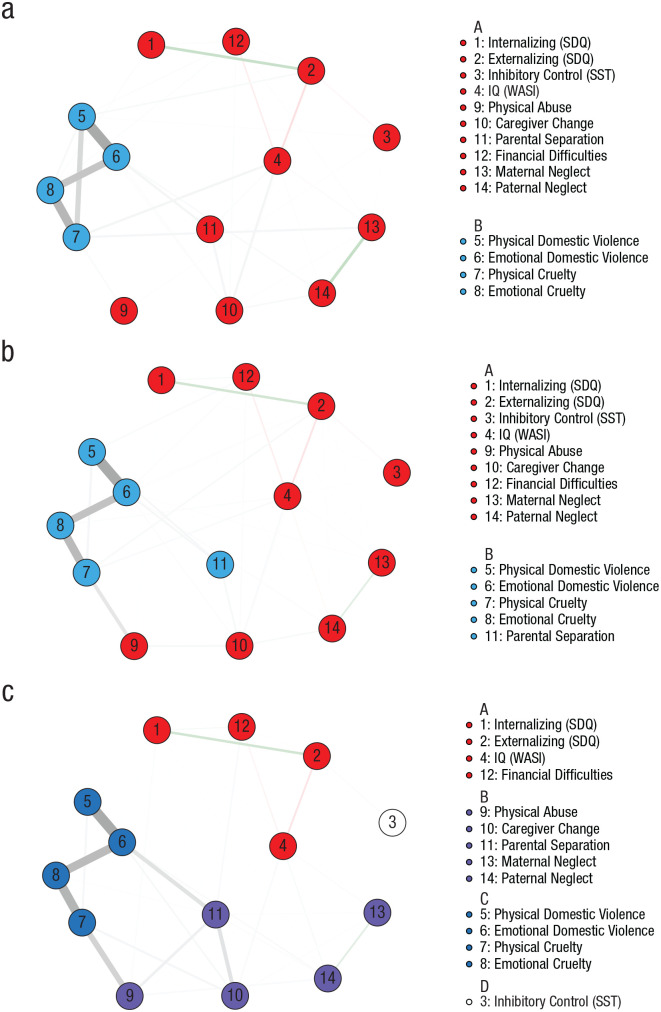
Subnetworks of adversities and adolescent emotional functioning and cognition. The three subnetworks correspond to adverse experiences that occurred between (a) birth and 1.5 years, (b) 1.5 to 5 years, and (c) 5 to 7 years. Nodes are arranged according to the average layout across the three networks. The color of each node denotes its group membership, and the thickness of each edge denotes its strength. Green and red edges correspond to positive and negative relationships of conditional dependence, whereas gray edges indicate relationships of conditional dependence with binary variables. SDQ = Strengths and Difficulties Questionnaire; SST = stop-signal task; WASI = Wechsler Abbreviated Scale of Intelligence.

The partitioning algorithm identified differences in the network structure on the basis of the timing of early adversity, with the optimal split points at 1.5 and 5 years old. The three resultant subnetworks are shown in Figure 4. Although clustering varied across the subnetworks, measures of emotional functioning and cognition consistently clustered with measures of deprivation, apart from inhibitory control in the third subnetwork. The range of forms of deprivation in this cluster narrowed across childhood, particularly in the transition from early childhood (Fig. 4b) to middle childhood (Fig. 4c). Analysis of the full sample produced similar clusters (see Fig. S4).

Modularity was low across the subnetworks (*Q* = 0.27, *Q* = 0.22, *Q* = 0.25). The node-strength and bridge-strength indices of the subnetworks can be found in Figure S5. Node and bridge strength were highly stable, with CS values of 0.94, 0.97, 0.90 and 0.91, 0.97, 0.80 respectively (Fig. S6). Given the low modularity of the clusters, we did not perform formal network comparisons.

### Exploratory network analyses

**Fig. 5.**
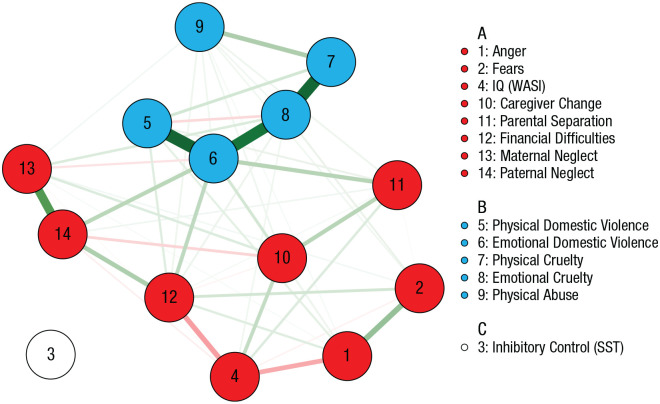
Network of summed adversities across childhood, adolescent anger and fear reactivity, and adolescent cognition. The color of each node denotes its group membership, and the thickness of each edge denotes its strength. Green and red edges correspond to positive and negative relationships of conditional dependence, respectively. SST = stop-signal task; WASI = Wechsler Abbreviated Scale of Intelligence.

One critique of our approach is that the SDQ measures not only emotional dysregulation but also psychopathology, which previous research has linked to both deprivation and threat (Miller et al., 2018). Therefore, in a deviation from our preregistration, we sought to more specifically target emotional functioning by recalculating our primary network model (shown in Fig. 2) with other maternal questionnaire measures from the same time point—namely, we substituted externalizing symptoms with a measure of anger reactivity (“temper tantrums or hot tempers”), and internalizing symptoms for a measure of fear reactivity (“many fears, is easily scared”). As shown in Figure 5, these measures also clustered with experiences of deprivation. Overall, the network shows the same pattern of clustering as our original analysis, with the exception that inhibitory control became a disconnected node because of weaker associations with anger and fear than with externalizing and internalizing symptoms.

A second potential weakness of our preregistered analysis is that it does not control for the moderating effects of demographic characteristics. Although the birth cohort is relatively homogenous (Boyd et al., 2013), controlling for such characteristics would guard against spurious associations. We therefore deviated from our preregistration and recalculated the networks including child sex, month of birth, birth weight, ethnicity, and maternal social class as moderators in each node-wide regression. The overall network, found in Figure S8 in the Supplemental Material, showed identical clustering to that of Figure 2. In the network of adversities alone, the deprivation cluster was identical to that of Figure 3, but the threat cluster split into a pair of domestic-violence measures and a trio of abuse measures (see Fig. S9 in the Supplemental Material).

## Discussion

We tested the dimensionality of early adverse experiences. Previous attempts to establish specific links between deprivation and threat and their respective outcomes have not accounted for the complexity of interrelations between these variables, a limitation overcome by using network analysis (Sheridan et al., 2020). We increased the sample size and used longitudinal, rather than retrospective, measures of adversity. We found evidence for a distinction between deprivation and threat in the model of adversities alone and identified a broad association between early experiences of deprivation and later cognition and emotion that narrowed across infancy, early childhood, and middle childhood. However, we were unable to identify a specific cluster between early experiences of threat and subsequent emotional regulation in adolescence with our measures.

Contrary to our first hypothesis, our findings do not fully support the dimensional model of adversity and psychopathology. We had expected threat-related adversities to cluster with externalizing and internalizing problems, as previous work identified a direct path to these symptoms from threat but not deprivation (Miller et al., 2018). We then expected deprivation-related adversities to cluster with measures of cognition because of the proposed limits they impose on associative learning and cognitive development (McLaughlin et al., 2017). Instead, while a delineation of deprivation and threat emerged in a network of adversities alone, when we introduced measures of adolescent cognitive and emotional functioning into the network, they all clustered with deprivation. Furthermore, the modularity index of the network was low, indicating a weak separation between clusters. Consequently, our results cohere well with previous work indicating that a single adversity factor, which draws heavily on deprivation-related features of the early environment, can explain much of the variance in later cognition and behavior (Bignardi et al., 2022). Because deprivation involves not only a lack of material resources but also inadequate psychosocial care (King et al., 2019), this dimension may capture a broader range of important features of the environment of a child.

As predicted, the network structure of early adversity and adolescent emotional functioning and cognition varied by the timing of the adversity. Our recursive partitioning analysis split the data into infancy, early childhood, and middle childhood, a finding that aligns well with previous research on timing (e.g., Dunn et al., 2020). In each segment of childhood, measures of adolescent cognition and emotional functioning clustered with progressively fewer measures of early deprivation: all forms during infancy, all but parental separation during early childhood, and only financial difficulties during middle childhood. Because various sensitive periods exist across childhood for the development of neural and behavioral characteristics (Knudsen, 2004), the narrowing of the deprivation cluster may reflect the disproportionate impact of specific forms of adversity at earlier stages of development. Although we did not consider the duration or severity of adversity, and therefore cannot disentangle these features from age, our interpretation is strengthened by controlling for exposure to adversity at other stages of childhood. Furthermore, our results are consistent with a large body of previous work linking both cognitive difficulties and externalizing and internalizing problems to early caregiving instability, low socioeconomic status, and neglect (Almas et al., 2020; Fields et al., 2021; Geoffroy et al., 2016; Lansford et al., 2019; Manly et al., 2013; von Stumm & Plomin, 2015).

Several key features of our study allowed us to extend the work of Sheridan et al. (2020). First, we used prospective measures of adversity rather than relying on retrospective self-report. Because concurrently measuring childhood adversity and its sequelae in early adulthood can artificially inflate associations between these variables (Newbury et al., 2018), our clusters may more accurately capture the relationship between early adversity and later cognition and emotion. Second, our use of a birth cohort enabled us to increase the sample size by about tenfold, improving the robustness of our analysis and its population representativeness. Third, our consideration of additional psychosocial forms of deprivation allowed us to confirm the breadth of this dimension. Finally, our consideration of the developmental timing of adversity allowed us to uncover underlying heterogeneity in the network.

Another important difference between our study and Sheridan et al.’s (2020) work is our inclusion of internalizing and externalizing problems as measured by the SDQ, rather than direct measures of emotional reactivity (such as a Stroop task). We chose this approach to assess broader possible effects of early adversity. However, the SDQ indexes not only emotional functioning but also psychopathology, which the dimensional model traces back to early experiences of both deprivation and threat (Miller et al., 2018, 2021). Our use of internalizing and externalizing symptoms may therefore have enabled us to compare the breadth and strength of the links between early deprivation and threat, and later well-being, but not their specificity. To explore this further, we constructed another network in which internalizing and externalizing symptoms were replaced with specific questions about adolescent anger and fear reactivity. These additional measures also clustered with deprivation, supporting our original findings. It is possible that our questionnaire data may be less sensitive and reliable than the cognitive tasks included in our networks and that this difference could mask our ability to detect specific relationships with types of adversity. To test this possibility, we would need a large-scale prospective longitudinal data set that incorporates a validated performance-based assessment of emotional regulation.

Several limitations of our study point to avenues for future research. Because ALSPAC includes few participants from racial and ethnic minorities, replicating our findings in a more diverse sample would confirm their generalizability. Similarly, ALSPAC suffers from substantial attrition, and although our imputation and analysis of the full sample yielded consistent results, our findings would benefit from replication in a longitudinal study with greater retention. Third, because the accuracy of discrete clustering analyses depends on the degree of separation between clusters, and our networks were weakly modular, future analyses should consider using a “fuzzy” clustering approach (Dalmaijer et al., 2022). Finally, the inclusion of other forms of early adversity in the network could uncover additional dimensions of experience, such as unpredictability or harshness (Ellis et al., 2009).

In summary, adverse experiences tend to co-occur in the population as deprivation or threat, and experiences that fit into the former dimension are broadly linked to later cognitive and emotional difficulties.

## Supplemental Material

sj-pdf-1-pss-10.1177_09567976221101045 – Supplemental material for Testing Deprivation and Threat: A Preregistered Network Analysis of the Dimensions of Early Adversity Supplemental material, sj-pdf-1-pss-10.1177_09567976221101045 for Testing Deprivation and Threat: A Preregistered Network Analysis of the Dimensions of Early Adversity by Sofia Carozza, Joni Holmes and Duncan E. Astle in Psychological Science

## Transparency

*Action Editor:* Leah Somerville

*Editor:* Patricia J. Bauer

*Author Contributions*

All authors designed the study. S. Carozza completed the data analysis and wrote the original draft under the supervision of J. Holmes and D. Astle. All the authors revised the manuscript and approved the final version for submission.

**Open Practices:** The code necessary to reproduce the analyses has been made publicly available via OSF and can be accessed at https://osf.io/y6f2c/. The design and analysis plans for the study were preregistered on AsPredicted at https://aspredicted.org/dp8r5.pdf. This article has received the badges for Open Materials and Preregistration. More information about the Open Practices badges can be found at http://www.psychologicalscience.org/publications/badges.
