# Supplementary material for: Testing Deprivation and Threat: A Preregistered Network Analysis of the Dimensions of Early Adversity
Source: Psychol Sci. 2022 Sep 8;33(10):1753–66. doi: 10.1177/09567976221101045 (PMC13020948; doi:10.1177/09567976221101045)
Supplement: sj-pdf-1-pss-10.1177_09567976221101045 – Supplemental material for Testing Deprivation and Threat: A Preregistered Network Analysis of the Dimensions of Early Adversity [file sj-pdf-1-pss-10.1177_09567976221101045.pdf]

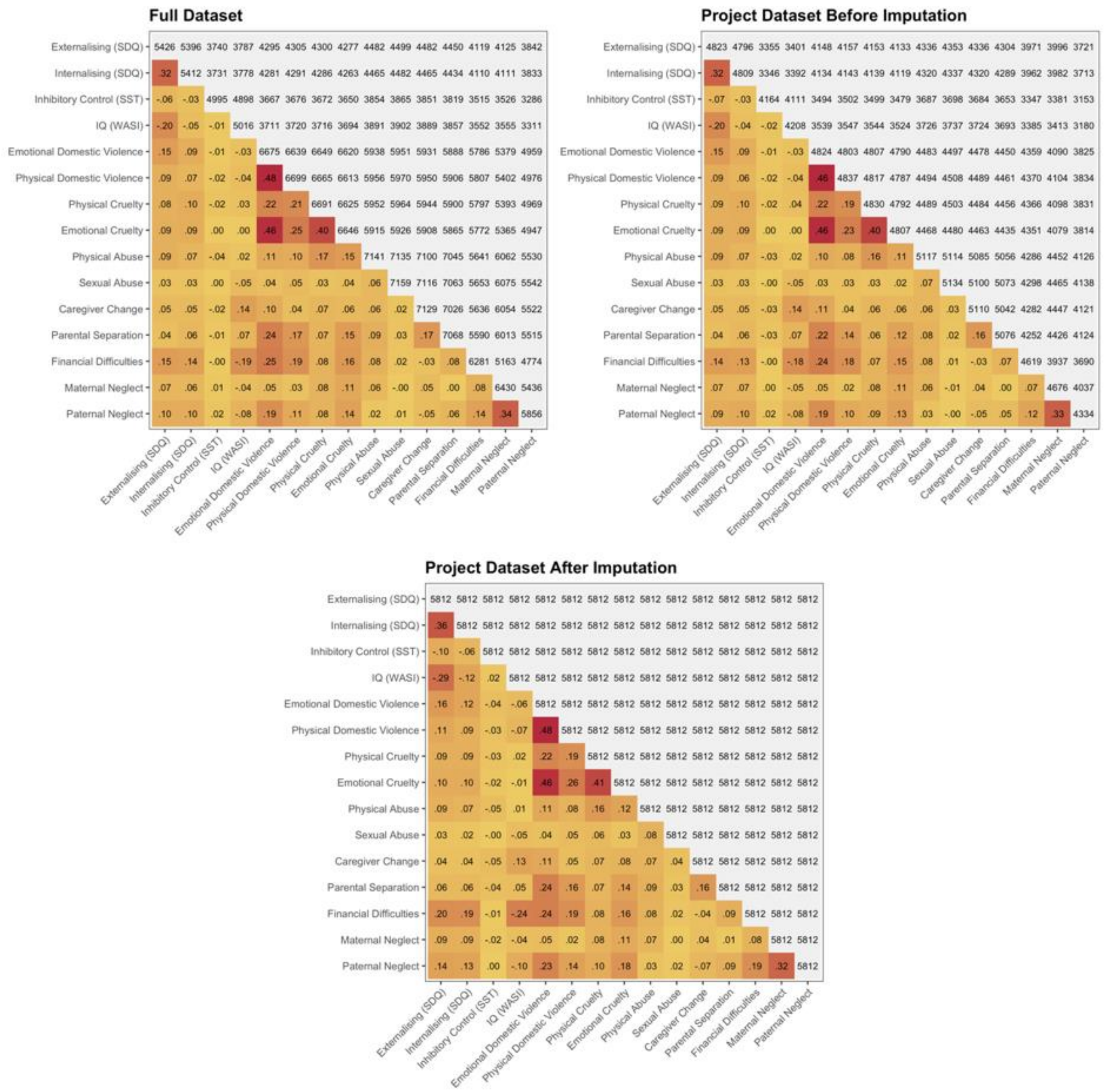

Figure S1. Spearman correlations of network variables across samples. The number of pairwise complete observations is on the upper diagonal, and the correlation coefficient on the lower diagonal. Red indicates stronger associations. SDQ = Strengths and Difficulties Questionnaire; SST = Stop Signal Task; WASI = Wechsler Abbreviated Scale of Intelligence.

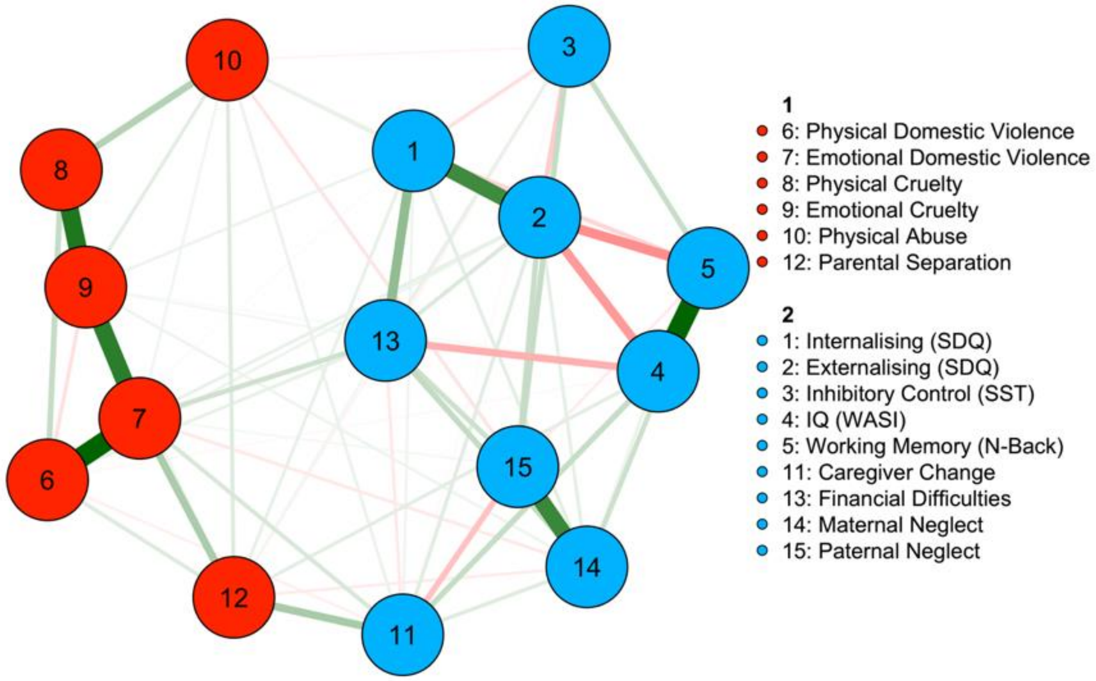

Figure S2. Full-sample network of summed adversities across childhood and adolescent emotional functioning and cognition. All participants and variables were included, regardless of exclusion criteria. The colour of each node denotes its group membership, and the thickness of each edge denotes its strength. Green and red edges correspond to positive and negative relationships of conditional dependence, respectively. SDQ = Strengths and Difficulties Questionnaire; SST = Stop Signal Task; WASI = Wechsler Abbreviated Scale of Intelligence.

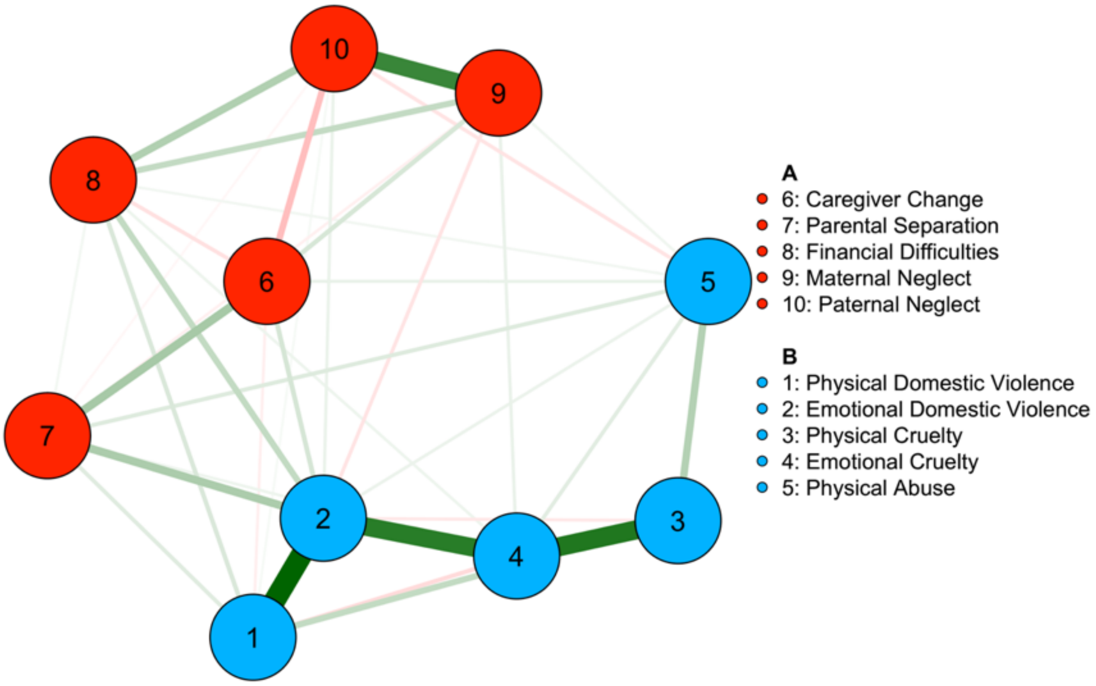

Figure S3. Full-sample network of summed adversities across childhood. All participants were included, regardless of exclusion criteria. The colour of each node denotes its group membership, and the thickness of each edge denotes its strength. Green and red edges correspond to positive and negative relationships of conditional dependence, respectively. SDQ = Strengths and Difficulties Questionnaire; SST = Stop Signal Task; WASI = Wechsler Abbreviated Scale of Intelligence.

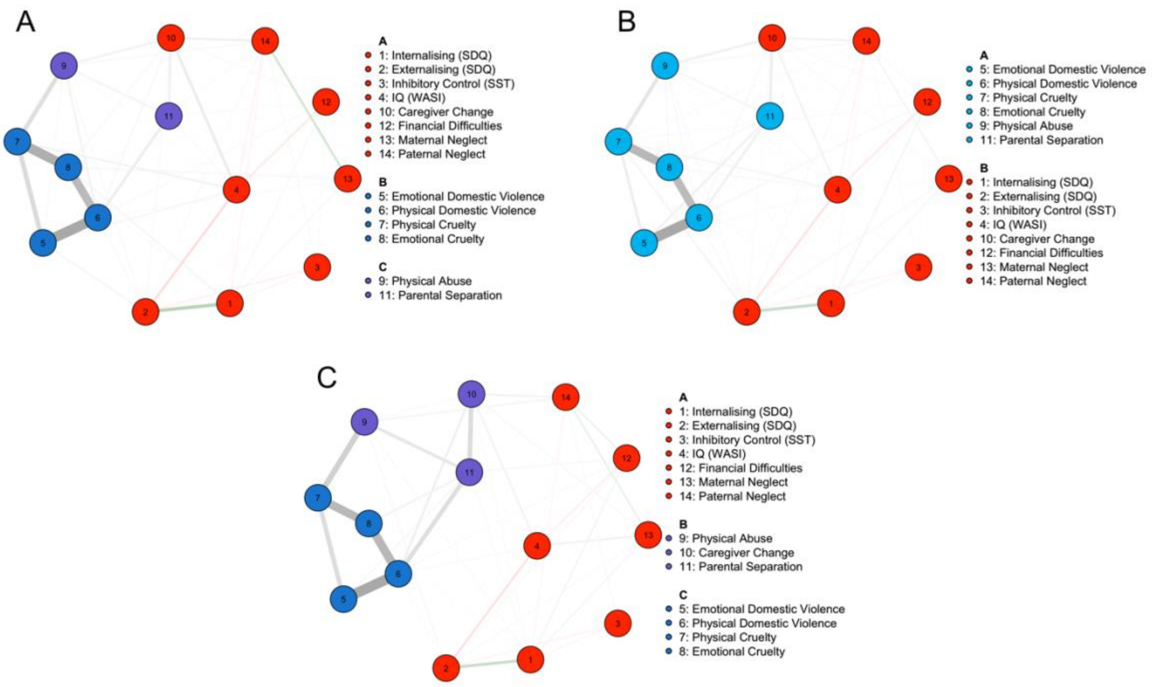

Figure S4. Full-sample subnetworks of adversities and adolescent emotional functioning and cognition. All participants and variables were included, regardless of exclusion criteria. The three subnetworks correspond to adverse experiences that occurred between (A) birth and 1.5 years, (B) 1.5 to 5 years, and (C) 5 to 7 years of age. Nodes are arranged according to the average layout across the three networks. The colour of each node denotes its group membership, and the thickness of each edge denotes its strength. Green and red edges correspond to positive and negative relationships of conditional dependence, while grey edges indicate relationships of conditional dependence with binary variables. SDQ = Strengths and Difficulties Questionnaire; SS = Stop Signal Task; WASI = Wechsler Abbreviated Scale of Intelligence.

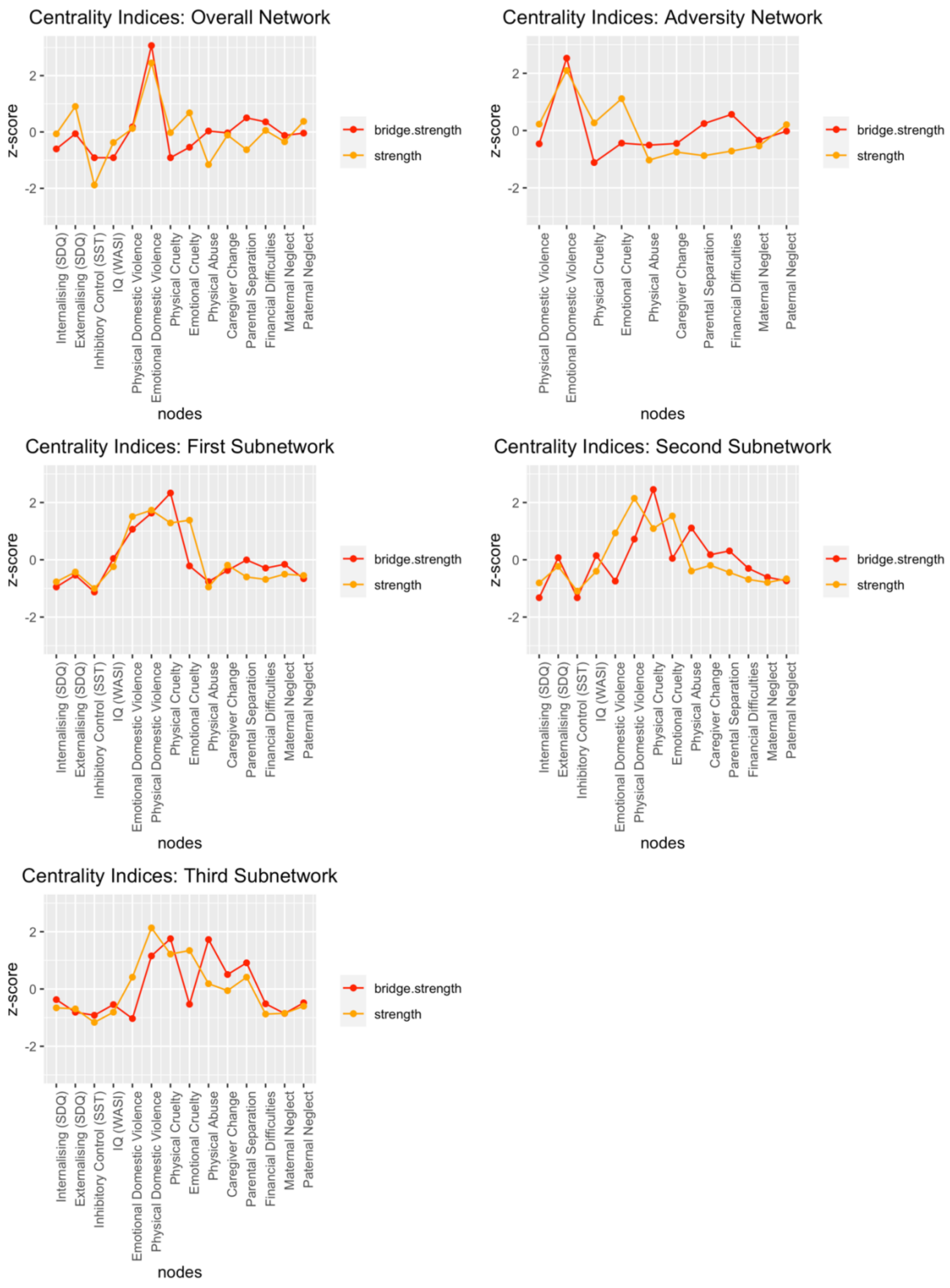

Figure S5. Centrality measures across networks. SDQ = Strengths and Difficulties Questionnaire; SST = Stop Signal Task; WASI = Wechsler Abbreviated Scale of Intelligence.

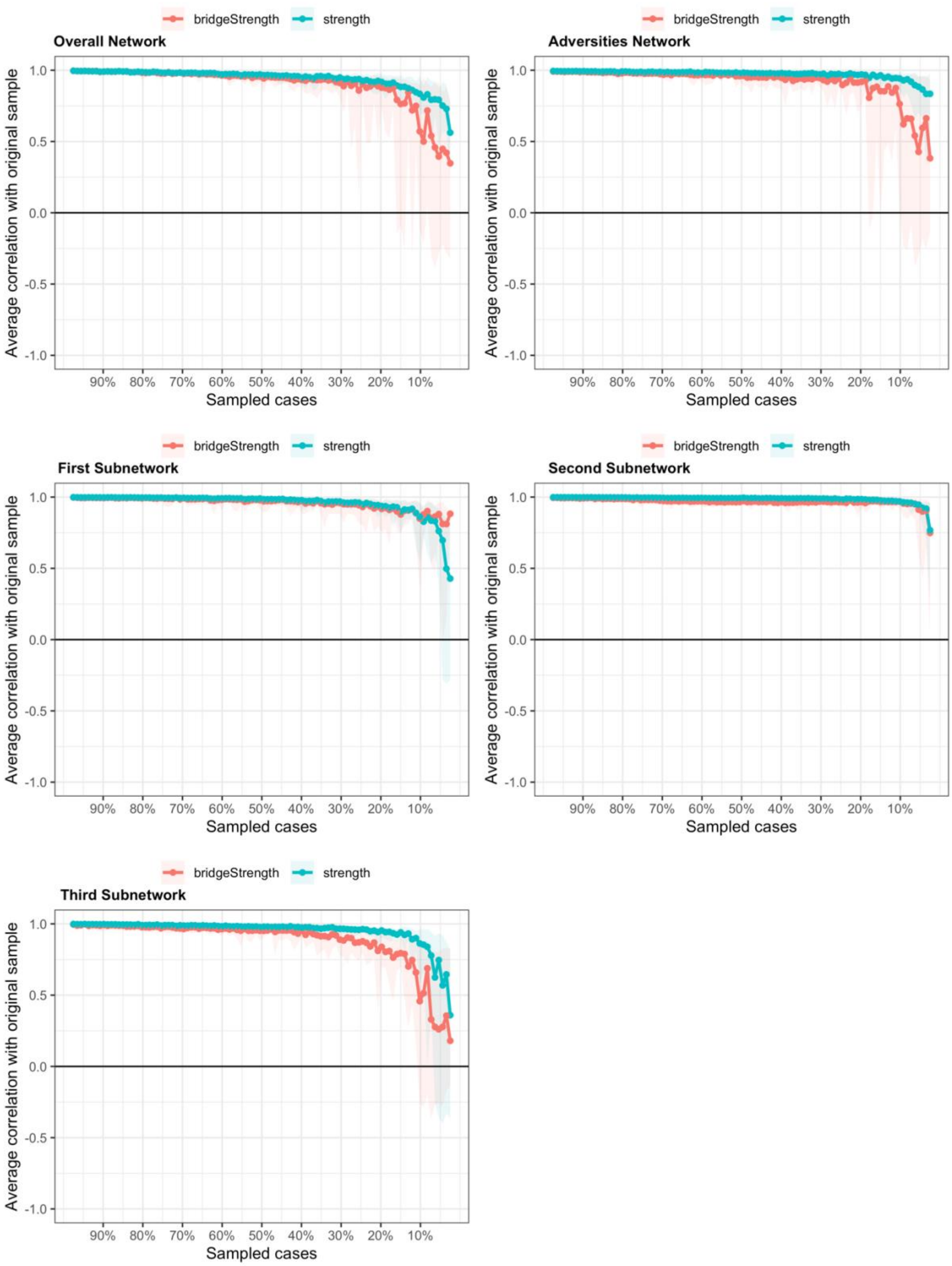

Figure S6. Bootstrapped stability of estimated parameters across networks.

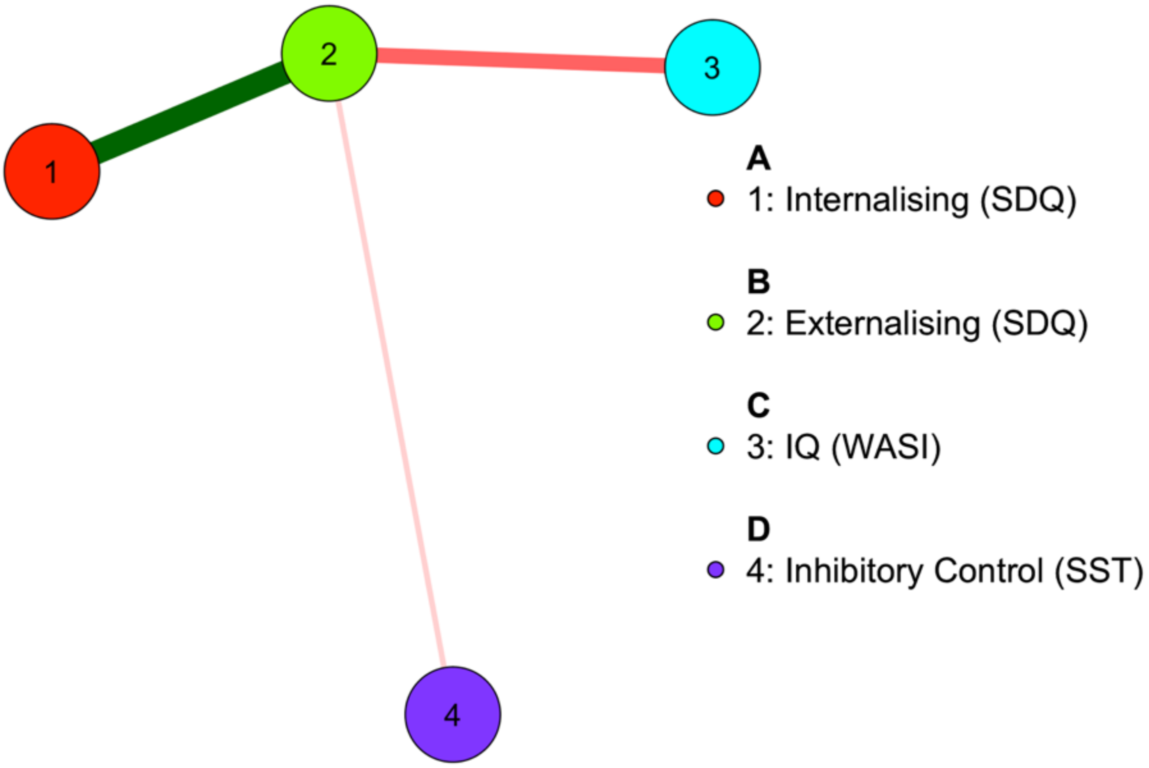

Figure S7. Network of adolescent emotional functioning and cognition. The colour of each node denotes its group membership, and the thickness of each edge denotes its strength. Green and red edges correspond to positive and negative relationships of conditional dependence, respectively. SDQ = Strengths and Difficulties Questionnaire; SST = Stop Signal Task; WASI = Wechsler Abbreviated Scale of Intelligence.

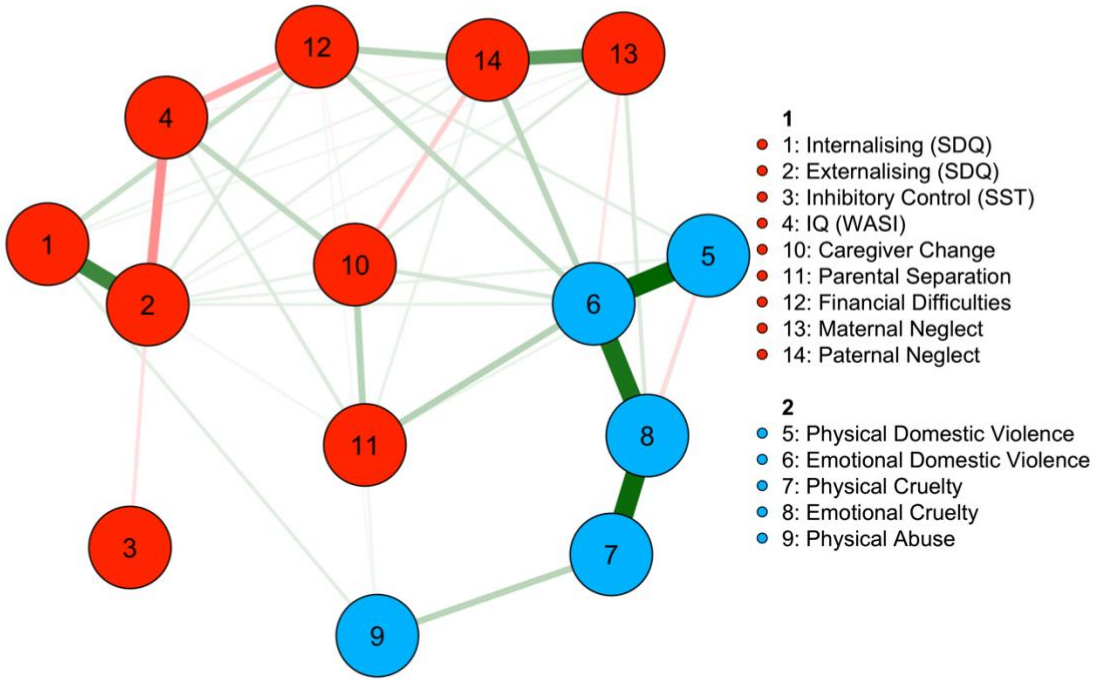

Figure S8. Network of summed adversities across childhood and adolescent emotional functioning and cognition. The analysis was controlled for child sex, month of birth, birthweight, ethnicity, and maternal social class, by implementing these variables as moderators in each node-wise regression. The colour of each node denotes its group membership, and the thickness of each edge denotes its strength. Green and red edges correspond to positive and negative relationships of conditional dependence, respectively. SDQ = Strengths and Difficulties Questionnaire; SST = Stop Signal Task; WASI = Wechsler Abbreviated Scale of Intelligence.

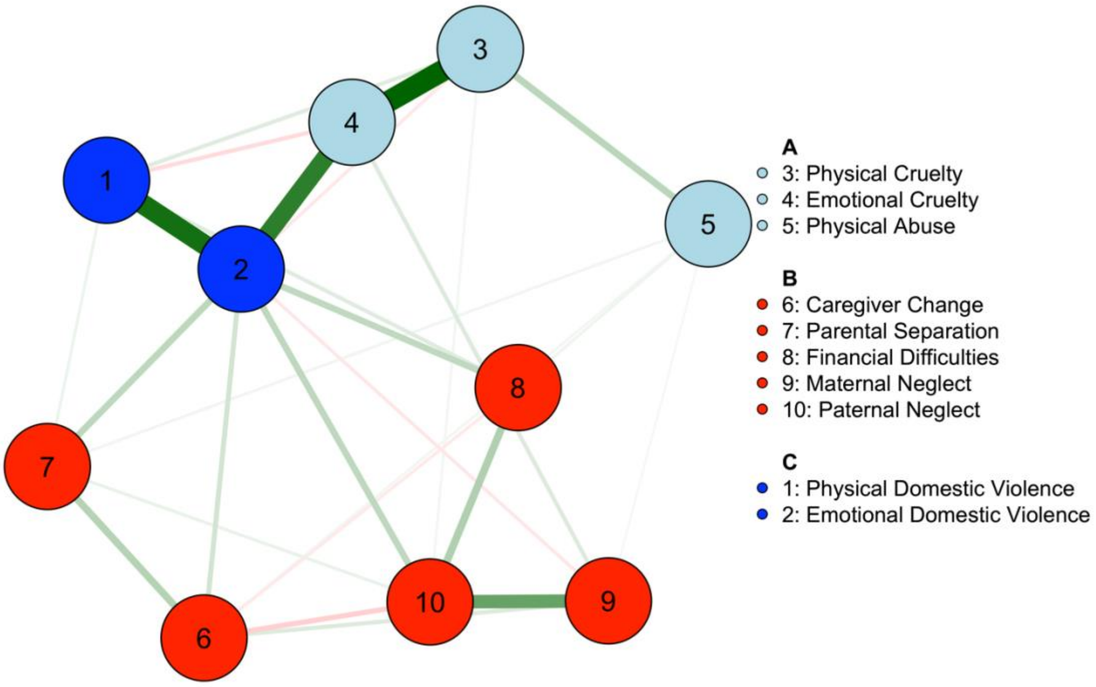

Figure S9. Network of summed adversities across childhood. The analysis was controlled for child sex, month of birth, birthweight, ethnicity, and maternal social class, by implementing these variables as moderators in each node-wise regression. The colour of each node denotes its group membership, and the thickness of each edge denotes its strength. Green and red edges correspond to positive and negative relationships of conditional dependence, respectively. SDQ = Strengths and Difficulties Questionnaire; SST = Stop Signal Task; WASI = Wechsler Abbreviated Scale of Intelligence.

Table S1
Data Collection Timeline by Variable

Variable	Time points of assessment
Physical domestic violence	18 m, 33 m, 45 m, 61 m, 73 m
Emotional domestic violence	18 m, 33 m, 45 m, 61 m, 73 m
Physical cruelty	18 m, 33 m, 45 m, 61 m, 73 m
Emotional cruelty	18 m, 33 m, 45 m, 61 m, 73 m
Sexual abuse	21 m, 30 m, 42 m, 57 m, 81 m
Physical abuse	21 m, 30 m, 42 m, 57 m, 81 m
Change in primary caregiver	21 m, 30 m, 42 m, 57 m, 81 m
Prolonged separation from parent	21 m, 30 m, 42 m, 57 m, 81 m
Financial difficulties score	8 m, 21 m, 33 m, 61 m, 85 m
Maternal caregiver neglect score	6 m, 18 m, 38 m, 57 m, 81 m
Paternal caregiver neglect score	6 m, 18 m, 38 m, 57 m, 81 m
IQ (WASI)	15.5 y
Inhibitory control (Stop Signal Task)	15.5 y
Working memory (N-Back Task)	17 y
Internalizing behaviours (SDQ)	16 y
Externalising behaviours (SDQ)	16 y

Table S2
Questions Used to Assess Adverse Experiences

Binary exposures	Questions
Physical domestic violence	A positive response to "Your partner was physically cruel to you since [previous time point]"
Emotional domestic violence	A positive response to "Your partner was emotionally cruel to you since [previous time point]."
Physical cruelty	A positive response to "Your partner was physically cruel to your children since [age at previous time point]" or "You were physically cruel to your children since [age at previous time point]."
Emotional cruelty	A positive response to "Your partner was emotionally cruel to your children since [age at previous time point]" or "You were emotionally cruel to your children since [age at previous time point]."
Sexual abuse	A positive response to "Was the child sexually abused since [age at previous time point]?"
Physical abuse	A positive response to "Was the child physically hurt by anyone since [age at previous time point]?"
Change in caregiver	A positive response to "Child was separated from his/her mother since [age at previous time point]" or "Child was separated from his/her father since [age at previous time point]."
Separation from parent	A positive response to "Child changed carer since [age at previous time point]."

Continuous exposures	Components of composite score	
Financial difficulties	Difficulty affording food	
	Difficulty affording clothing	
	Difficulty affording heating	
	Difficulty affording rent or mortgage	
	Difficulty affording items for child	
Maternal caregiver neglect and paternal caregiver neglect	Time point 1	Mum/partner plays with child
		Mum/partner sings to child
		Mum/partner shows child picture books
		Mum/partner and child play with toys
		Mum/partner cuddles child
		Mum/partner physically plays with child
		Mum/partner takes child for walks
		Partner bathes child
		Partner feeds child
	Time point 2	Mum/partner bathes child
		Mum/partner feeds child
		Mum/partner sings to child
		Mum/partner reads to child
		Mum/partner plays with toys with child
		Mum/partner cuddles child
		Mum/partner plays pat-a-cake etc with child
		Mum/partner has physical play with child
		Mum/partner takes child for walks
	Time point 3	Mum/partner bathes child
		Mum/partner feeds child
		Mum/partner sings to child
		Mum/partner shows child pictures in book

	Mum/partner and child play with toys
	Mum/partner cuddles child
	Mum/partner has physical play with child
	Mum/partner takes child for walks
	Mum/partner puts child to bed
Time point 4	Mum/partner gives child a bath or shower
	Mum/partner makes things with child
	Mum/partner sings to child
	Mum/partner reads to child
	Mum/partner plays with toys with child
	Mum/partner cuddles child
	Mum/partner takes part in active play with child
	Mum/partner takes child to park or playground
	Mum/partner puts child to bed
	Mum/partner takes child swimming
	Mum/partner draws or paints with child
	Mum/partner prepares food for child
Time point 5	Mum/partner bathes child
	Mum/partner makes things with child
	Mum/partner sings to child
	Mum/partner reads to child
	Mum/partner plays with toys with child
	Mum/partner cuddles child
	Mum/partner does active play with child
	Mum/partner takes child to park/playground
	Mum/partner puts child to bed
	Mum/partner takes child swimming
	Mum/partner draws/paints with child
	Mum/partner prepares food for child
	Mum/partner takes child to classes
	Mum/partner takes child shopping
	Mum/partner takes child to watch sports
	Mum/partner does homework with child
	Mum/partner has conversations with child
	Mum/partner helps child prepare for school

Table S3

Descriptive Statistics of Adolescent Outcomes and Summed Childhood Adversities by Sample.

	Full sample (N = 13957)	Pre-imputation (N = 5812)	Post-imputation (N = 5812)
Externalising			
min	0	0	0
max	18	18	18
mean ± sd	11.94 ± 12.86	11.27 ± 12.49	11.77 ± 12.74
Internalising			
min	0	0	0
max	17	17	17
mean ± sd	11.94 ± 12.86	11.27 ± 12.49	11.77 ± 12.74
Inhibitory Control			
min	0	14	14
max	32	32	32
mean ± sd	11.94 ± 12.86	11.27 ± 12.49	11.77 ± 12.74
Intelligence			
min	55	56	56
max	136	131	131
mean ± sd	94.35 ± 13.08	95.05 ± 12.81	94.72 ± 11.28
Financial Difficulties			
min	0	0	0
max	70	70	70
mean ± sd	11.94 ± 12.86	11.27 ± 12.49	11.77 ± 12.74
Maternal Neglect			
min	5	5	5
max	13.14	13.14	13.14
mean ± sd	7.68 ± 1.15	7.67 ± 1.13	7.68 ± 1.13
Paternal Neglect			
min	5.22	5.89	5.72
max	20.22	20.22	20.22
mean ± sd	10.65 ± 2.07	10.61 ± 2.04	10.83 ± 2.20
	N (%)	N (%)	N (%)
Sex			
Male	7,206 (52%)	2,835 (49)	2,835 (49)
Female	6,751 (48)	2,977 (51)	2,977 (51)
Emotional Domestic Violence			
0	5,103 (76)	3,759 (78)	4,501 (77)
1	766 (11)	520 (11)	657 (11)
2	407 (6)	273 (6)	330 (6)
3	218 (3)	150 (3)	182 (3)
4	101 (2)	60 (1)	70 (1)
5	80 (1)	62 (1)	72 (1)
Physical Domestic Violence			

	Full sample (N = 13957)	Pre-imputation (N = 5812)	Post-imputation (N = 5812)
0	6,181 (92)	4,520 (93)	5,402 (93)
1	318 (5)	196 (4)	255 (4)
2	133 (2)	77 (2)	98 (2)
3	43 (1)	29 (1)	36 (1)
4+	24 (0)	15 (0)	21(0)
Emotional Cruelty			
0	5,917 (89)	4,295 (89)	5,192 (89)
1	453 (7)	307 (6)	371 (6)
2	160 (2)	120 (2)	152 (3)
3	64 (1)	42 (1)	49 (1)
4	38 (1)	31 (1)	34 (1)
5	14 (0)	12 (0)	14 (0)
Physical Cruelty			
0	6,416 (96)	4,623 (96)	5,571 (96)
1	193 (3)	146 (3)	168 (3)
2	48 (1)	35 (1)	42 (1)
3	23 (0)	20 (0)	24 (0)
4+	11 (0)	6 (0)	7 (0)
Physical Abuse			
0	6,336 (89)	4,549 (89)	5,169 (89)
1	614 (9)	425 (8)	481 (8)
2	137 (2)	101 (2)	118 (2)
3	42 (1)	32 (1)	34 (1)
4+	12 (0)	10 (0)	10 (0)
Sexual Abuse			
0	7,134 (100)	5,117 (100)	5,791 (100)
1+	25 (0)	17 (0)	21 (0)
Caregiver Change			
0	5,737 (80)	4,028 (79)	4,602 (79)
1	920 (13)	701 (14)	798 (14)
2	339 (5)	268 (5)	292 (5)
3	102 (1)	86 (2)	92 (2)
4+	31 (0)	27 (1)	28 (0)
Parental Separation			
0	3,870 (55)	2,790 (55)	3,198 (55)
1	1,654 (23)	1,193 (24)	1,369 (24)
2	884 (13)	629 (12)	732 (13)
3	476 (7)	337 (7)	375 (6)
4	148 (2)	103 (2)	113 (2)
5	36 (1)	24 (0)	25 (0)

** This may include zero.*

Note. The sum of each adversity measure was taken across five time points, such that binary exposures (e.g. emotional domestic violence) are quantified by the number of times they occurred across childhood and continuous exposures (e.g. financial difficulties) are quantified by their total sum across childhood. Cells have been collapsed where exact cell counts less than 5 could be inferred. “Full sample” = all participants in the core sample of the Avon Longitudinal Study of Parents and Children (ALSPAC) who were alive at one year of age; “Pre-imputation” = participants included in the present study before the imputation of missing data; “Post-imputation” = participants included in the present study after the imputation of missing data.

Table S4:
Adversity Measures by Individual Time Point

	Time 1 (N = 5812)	Time 2 (N = 5812)	Time 3 (N = 5812)	Time 4 (N = 5812)	Time 5 (N = 5812)
	N (%)	N (%)	N (%)	N (%)	N (%)
Emotional Domestic Violence					
0	5,650 (97)	5,673 (98)	5,674 (98)	5,709 (98)	5,708 (98)
1	162 (3)	139 (2)	138 (2)	103 (2)	104 (2)
Physical Domestic Violence					
0	5,158 (89)	5,249 (90)	5,398 (93)	5,334 (92)	5,418 (93)
1	654 (11)	563 (10)	414 (7)	478 (8)	394 (7)
Emotional Cruelty					
0	5,605 (96)	5,595 (96)	5,636 (97)	5,573 (96)	5,623 (97)
1	207 (4)	217 (4)	176 (3)	239 (4)	189 (3)
Physical Cruelty					
0	5,733 (99)	5,747 (99)	5,742 (99)	5,734 (99)	5,751 (99)
1	79 (1)	65 (1)	70 (1)	78 (1)	61 (1)
Physical Abuse					
0	5,678 (98)	5,597 (96)	5,651 (97)	5,620 (97)	5,652 (97)
1	134 (2)	215 (4)	161 (3)	192 (3)	160 (3)
Caregiver Change					
0	5,381 (93)	5,365 (92)	5,434 (93)	5,423 (93)	5,684 (98)
1	431 (7)	447 (8)	378 (7)	389 (7)	128 (2)
Parental Separation					
0	4,697 (81)	4,667 (80)	4,704 (81)	5,091 (88)	5,366 (92)
1	1,115 (19)	1,145 (20)	1,108 (19)	721 (12)	446 (8)
Financial Difficulties					
min	0	0	0	0	0
max	15	15	15	15	15
mean ± sd	2.80 ± 3.39	2.69 ± 3.35	2.61 ± 3.28	2.04 ± 2.86	1.63 ± 2.51
Maternal Neglect					
min	7	9	9	12	18
max	17	30	34	37	82
mean ± sd	8.51 ± 1.53	12.54 ± 3.27	11.25 ± 2.57	17.97 ± 3.51	41.75 ± 6.97
Paternal Neglect					
min	9	9	9	12	18
max	29	45	36	46	90
mean ± sd	15.43 ± 3.77	20.82 ± 6.19	15.14 ± 4.51	23.65 ± 5.78	56.77 ± 10.42

** This may include zero*

Note. Assessments in Time 1 covered birth to 18 months of age; Time 2 covered 18 to 33 months of age; Time 3 covered 33 to 45 months of age; Time 4 covered 45 through 61 months of age; Time 5 covered 61 through 85 months of age.
